# cAMP potentiates InsP_3_-induced Ca^2+ ^release from the endoplasmic reticulum in blowfly salivary glands

**DOI:** 10.1186/1472-6793-8-10

**Published:** 2008-05-20

**Authors:** Ruth Schmidt, Otto Baumann, Bernd Walz

**Affiliations:** 1 Institute of Biochemistry and Biology, Department of Animal Physiology, University of Potsdam, Karl-Liebknecht-Str. 24-25, 14476 Potsdam-Golm, Germany

## Abstract

**Background:**

Serotonin induces fluid secretion from *Calliphora *salivary glands by the parallel activation of the InsP_3_/Ca^2+ ^and cAMP signaling pathways. We investigated whether cAMP affects 5-HT-induced Ca^2+ ^signaling and InsP_3_-induced Ca^2+ ^release from the endoplasmic reticulum (ER).

**Results:**

Increasing intracellular cAMP level by bath application of forskolin, IBMX or cAMP in the continuous presence of threshold 5-HT concentrations converted oscillatory [Ca^2+^]_i _changes into a sustained increase. Intraluminal Ca^2+ ^measurements in the ER of β-escin-permeabilized glands with mag-fura-2 revealed that cAMP augmented InsP_3_-induced Ca^2+ ^release in a concentration-dependent manner. This indicated that cAMP sensitized the InsP_3 _receptor Ca^2+ ^channel for InsP_3_. By using cAMP analogs that activated either protein kinase A (PKA) or Epac and the application of PKA-inhibitors, we found that cAMP-induced augmentation of InsP_3_-induced Ca^2+ ^release was mediated by PKA not by Epac. Recordings of the transepithelial potential of the glands suggested that cAMP sensitized the InsP_3_/Ca^2+ ^signaling pathway for 5-HT, because IBMX potentiated Ca^2+^-dependent Cl^- ^transport activated by a threshold 5-HT concentration.

**Conclusion:**

This report shows, for the first time for an insect system, that cAMP can potentiate InsP_3_-induced Ca^2+ ^release from the ER in a PKA-dependent manner, and that this crosstalk between cAMP and InsP_3_/Ca^2+ ^signaling pathways enhances transepithelial electrolyte transport.

## Background

Calcium ions and cyclic AMP are ubiquitous intracellular messengers that regulate a plethora of cellular processes. Indeed, the stimulation of many non-excitable cells by neurotransmitters or hormones causes the parallel activation of the cAMP and the phosphoinositide signaling pathways [1,2]. The latter culminates in inositol 1,4,5-trisphosphate (InsP_3_)-induced Ca^2+ ^release through InsP_3 _receptor Ca^2+ ^channels (InsP_3_R) from the endoplasmic reticulum (ER) and an elevation in intracellular Ca^2+ ^concentration ([Ca^2+^]_i_). InsP_3_-induced Ca^2+ ^release with or without Ca^2+ ^entry from the extracellular space generates temporally and spatially coordinated Ca^2+ ^signals leading, in many cells, to intracellular Ca^2+ ^oscillations and waves [3-5]. Thus, Ca^2+ ^signals can be spatially compartmentalized and coded by amplitude, frequency, and/or shape: these parameters are important for the specificity of stimulus response coupling [5].

One way of controlling Ca^2+ ^signals can be achieved by cAMP, which has been shown to affect Ca^2+ ^signaling at multiple sites, e.g., at the level of InsP_3 _generation [6-8] and InsP_3_-induced Ca^2+ ^release from the ER. cAMP exerts its physiological effects through downstream effector proteins, either protein kinase A (PKA) or cAMP-specific guanine nucleotide exchange factors (cAMP-GEF) known as exchange proteins directly activated by cAMP (Epac) [9,10]. Upon activation by cAMP, PKA is able to phosphorylate all three subtypes of vertebrate InsP_3_R and thus to modulate InsP_3_-induced Ca^2+ ^release from the ER [1,11-17]. On the other hand, physiological evidence from pancreatic β cells indicates that Epac sensitizes Ca^2+^-induced Ca^2+ ^release (CICR) via InsP_3_-R in a cAMP-dependent manner [18].

Although we are beginning to understand the functional consequences of InsP_3 _receptor phosphorylation and its effects on InsP_3_-induced Ca^2+ ^release in some mammalian cell types, little knowledge is currently available about whether cAMP affects InsP_3_-induced Ca^2+ ^release in invertebrates [19]. Only a single InsP_3_R isoform is expressed in *Drosophila melanogaster *(DmInsP_3_R) [20,21] and *Caenorhabditis elegans *(CeInsP_3_R). InsP_3_R in both species share the main functional properties with mammalian InsP_3_R: InsP_3 _sensitivity, single channel conductance, gating, and a bell-shaped Ca^2+ ^dependence [22-24]. However, InsP_3_R phosphorylation has not been investigated in these species.

Since almost nothing is known regarding whether cAMP affects InsP_3_R function in invertebrates or its possible mode of action, we have studied this interaction in isolated salivary glands of the blowfly *Calliphora vicina*, a dipteran species closely related to *Drosophila*. *Calliphora *salivary glands secrete a KCl-rich saliva when stimulated with the neurohormone serotonin (5-hydroxytryptamine, 5-HT). 5-HT activates, in parallel, the cAMP and the phosphoinositide signaling cascade [25]. The latter leads to InsP_3_-induced Ca^2+ ^release from the ER and, at low 5-HT concentrations, to intracellular Ca^2+ ^oscillations through cyclical Ca^2+ ^release from and reuptake into the ER [26,27]. The Ca^2+ ^elevation activates transepithelial Cl^- ^transport, whereas the increase in cAMP level stimulates transepithelial K^+ ^transport [28-31]. The aim of the present study has been to investigate whether cAMP affects 5-HT-induced Ca^2+ ^signaling and InsP_3_-induced Ca^2+ ^release from the ER. We provide evidence that cAMP sensitizes the InsP_3_-sensitivity of InsP_3_-induced Ca^2+ ^release in a PKA-dependent manner.

## Results

### cAMP affects 5-HT-induced Ca^2+ ^signaling

**Figure 1:**
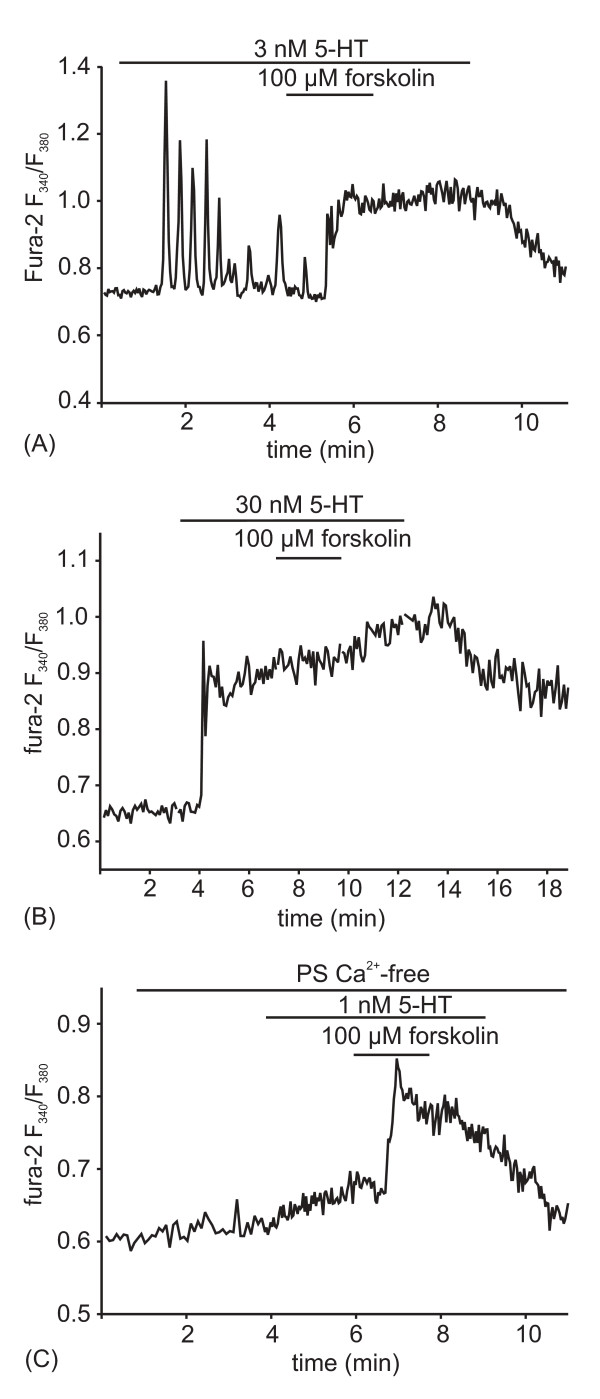
**Forskolin augments [Ca^2+^]_i _changes induced by low 5-HT concentrations in *Calliphora *salivary gland cells.** (A) Stimulation with 3 nM 5-HT produces intracellular Ca^2+ ^oscillations. Application of 100 μM forskolin converts oscillatory [Ca^2+^]_i _changes into a sustained increase (n = 8). (B) Stimulation of the gland with 30 nM 5-HT, a concentration that saturates fluid secretion, produces a biphasic Ca^2+ ^response consisting of an initial transient followed by a plateau of elevated [Ca^2+^]_i_. The sustained phase of elevated [Ca^2+^]_i _is not effected by forskolin (n = 4). (C) Application of a threshold concentration of 5-HT (1 nM) in Ca^2+^-free PS (0-Ca, 2 mM EGTA) increases [Ca^2+^]_i _just measurably without triggering Ca^2+ ^spikes. Additional application of 100 μM forskolin induces a transient Ca^2+ ^elevation, showing that forskolin augments 5-HT-induced Ca^2+ ^release, not Ca^2+ ^entry (n = 8).

Threshold concentrations of 5-HT (1–3 nM) induced intracellular Ca^2+ ^oscillations, whereas saturating 5-HT concentrations (> 30 nM) produced biphasic Ca^2+ ^responses that consisted of an initial transient followed by a plateau of elevated [Ca^2+^]_i _(Figs. 1A, B, and [26,27]). To test whether these two types of response patterns were affected by cAMP, we increased the intracellular cAMP by bath application of 10 mM cAMP, 100 μM IBMX, or 100 μM forskolin. These substances/concentrations had no effect on resting [Ca^2+^]_i _[33]. As shown in Fig. 1A, 3 nM 5-HT induced intracellular Ca^2+ ^oscillations, as described previously. Application of forskolin to the bath in the continuous presence of 3 nM 5-HT converted the oscillatory [Ca^2+^]_i _changes into a sustained increase (n = 8). Treatment with cAMP or IBMX had the same effect as forskolin at all tested preparations (cAMP, n = 7; IBMX, n = 5). Forskolin did not affect the sustained Ca^2+ ^elevation produced by 30 nM 5-HT (Fig. 1B), a concentration that saturates the rate of fluid secretion [34].

To determine whether the extra Ca^2+ ^increase produced by forskolin at low 5-HT concentrations was attributable to Ca^2+ ^influx from the extracellular space, we stimulated glands with a sub-threshold concentration of 5-HT (in order to prevent fast Ca^2+ ^store depletion [26]) and applied forskolin in Ca^2+^-free PS (no added Ca^2+^, 2 mM EGTA). As seen in Fig. 1C, 1 nM 5-HT was below the concentration that induced marked Ca^2+ ^oscillations (in Ca^2+^-containing PS), but application of 100 μM forskolin stimulated a transient Ca^2+ ^elevation even in the absence of extracellular Ca^2+^. Taken together, these results suggested that cAMP did not induce Ca^2+ ^influx but rather augmented Ca^2+ ^release from the ER produced by low 5-HT concentrations.

### cAMP augments InsP_3_-induced Ca^2+ ^release from the ER

Theoretically, there are two mechanisms for the release of Ca^2+ ^from the ER: the InsP_3_R and the ryanodine receptor Ca^2+ ^channel (RyR). Blowfly salivary glands, however, seem to lack RyR [26], leaving only the InsP_3_R as potential target for the cAMP pathway in order to enhance Ca^2+ ^release.

**Figure 2:**
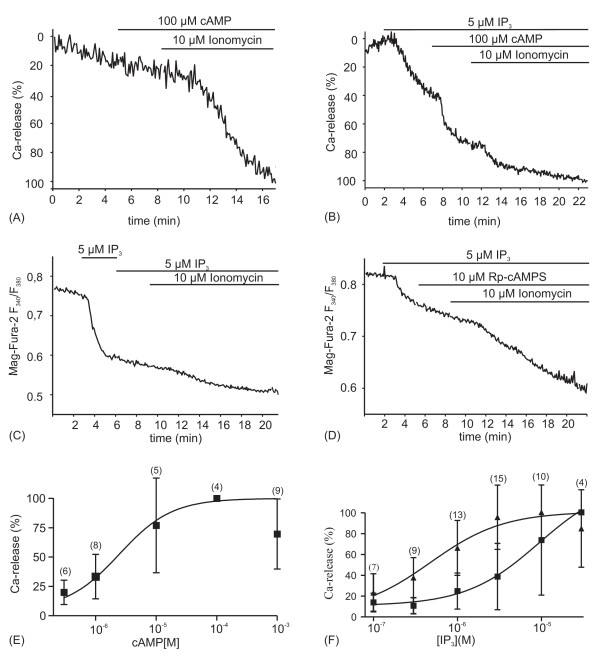
**cAMP augments InsP_3_-induced Ca^2+ ^release from β-escin permeabilized cells, as shown by intraluminal Ca^2+ ^measurements with Mag-Fura-2.** (A) cAMP does not induce Ca^2+ ^release from the ER (n = 4). (B) Application of 5 μM InsP_3 _induces Ca^2+ ^release from the ER and is augmented by 100 μM cAMP. (C, D) Ca^2+ ^release induced by 5 μM InsP_3 _is neither enhanced by application of fresh InsP_3 _solution (C) nor by mock stimulation with Rp-cAMPS (D). (E) Quantification of the cAMP-dependent augmentation of Ca^2+ ^release induced by 5 μM InsP_3 _from experiments as shown in B. 0% Ca^2+ ^release is the intraluminal Mag-fura-2 ratio at time zero of the recording; 100% Ca^2+ ^release is the fluorescence ratio after complete loss of intraluminal Ca^2+ ^following ionomycin application. A sigmoidal dose-response curve fitted to mean values (R^2 ^= 0.4) of the InsP_3_(+cAMP)-induced Ca^2+ ^release gives an EC_50, cAMP _of 2.6 μM. (F) Dose-response relationship for InsP_3_-induced Ca^2+ ^release in the presence (triangles) and absence (squares) of 100 μM cAMP. The leftward shift of the dose-response relationship indicates sensitization of InsP_3_-induced Ca^2+ ^release for InsP_3 _by cAMP. (E, F) The number of measurements for every data point is given in brackets. Means ± S.D.

To examine directly whether cAMP augmented InsP_3_-induced Ca^2+ ^release we studied Ca^2+ ^release from the ER by intraluminal Ca^2+ ^measurements with the low-affinity Ca^2+^-indicator dye Mag-fura-2. This dye accumulates within the ER and after β-escin permeabilization of the plasma membrane in an artificial "intracellular medium" (ICM) and loss of cytosolic dye, it monitors intraluminal Ca^2+ ^([Ca^2+^]_L_) [32,35,36]. Figures 2A and 2B show two representative original recordings of intraluminal Ca^2+ ^measurements. In order to facilitate the quantitative evaluation of this type of measurements, we converted Mag-fura-2 fluorescence ratios into a percentage scale, with 0% Ca^2+ ^release representing the intraluminal Mag-fura-2 ratio at time zero of the recording, and 100% Ca^2+ ^release representing the fluorescence ratio after the loss of intraluminal Ca^2+ ^following ionomycin application.

Application of 100 μM cAMP to the permeabilized gland tubules did not induce Ca^2+ ^release from the ER, whereas the Ca^2+^-ionophore ionomycin led to a dramatic loss in intraluminal Ca^2+ ^(Fig. 2A). Treatment with 5 μM InsP_3_, on the other hand, caused a partial Ca^2+ ^release, and the subsequent addition of 100 μM cAMP resulted in a further Ca^2+ ^release (Fig. 2B), indicating that cAMP had augmented InsP_3_-induced Ca^2+ ^release. In order to obtain the dose-response relationship for the effect of cAMP on InsP_3_-induced Ca^2+ ^release, the cAMP concentration was systematically varied, and Ca^2+ ^release (%) (Fig. 2E, squares) was measured after cAMP addition to ICM containing 5 μM InsP_3_. The sigmoidal dose-response curve fitted to the mean values of the InsP_3_(+cAMP)-induced Ca^2+ ^release gave a mean half maximal cAMP concentration (EC_50_) of 2.5 μM (Fig. 2E).

In order to exclude that the augmentation of InsP_3_-induced Ca^2+ ^release was not simply the result of the addition of fresh InsP_3_(+cAMP)-containing ICM, we superfused several preparations with InsP_3_(no cAMP)-containing ICM twice. A second InsP_3 _application never increased Ca^2+ ^release induced by a prior InsP_3 _application (Fig. 2C; n = 5). Moreover, mock stimulation with 10 μM (n = 5) or 100 μM (n = 5) 8-Br-Rp-cAMPS (a competitive antagonist of cAMP binding to PKA) had no significant effect on the InsP_3_-induced Ca^2+ ^release (Fig. 2D displays a representative original recording with 10 μM 8-Br-Rp-cAMP).

To determine whether cAMP increased the affinity of the InsP_3_R for InsP_3_, we examined Ca^2+ ^release induced by increasing InsP_3_-concentrations in the absence (Fig. 2F, squares) and presence of 100 μM cAMP (Fig. 2F, triangles). The two resulting dose-response curves indicated that cAMP increased the affinity of the InsP_3_R for InsP_3_, because cAMP shifted the dose-response curve to lower InsP_3 _concentrations by about one order of magnitude.

### Is the cAMP-dependent augmentation of InsP_3_-induced Ca^2+ ^release mediated by PKA or EPAC?

**Figure 3:**
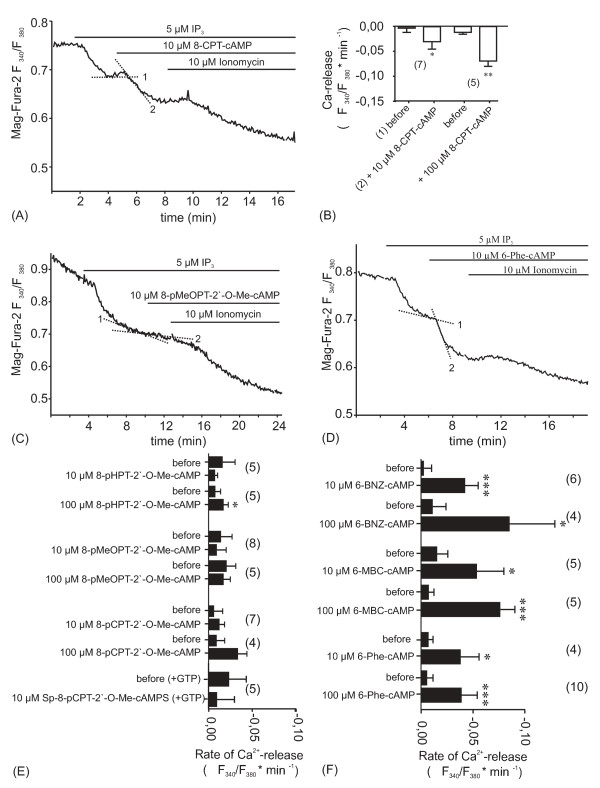
**InsP_3_-induced Ca^2+ ^release is augmented by PKA activators and not by Epac activators.** (A, C, D) Representative original recordings showing the effects of three cAMP analogs on InsP_3_-induced Ca^2+ ^release as recorded by intraluminal Ca^2+ ^measurements with Mag-Fura-2 in β-escin-permeabilized glands. (B, D, F) Summary of results obtained from experiments as illustrated in A, C and D. Ca^2+ ^release is displayed as the change in the rate of the Mag-Fura-2 fluorescence ratio (ΔF_340_/F_380_·min^-1^) before and after addition of a cAMP analog as shown in (A), dotted lines. (A, B) The PKA and Epac activator 8-CPT-cAMP augments InsP_3_-induced Ca^2+ ^release significantly in a concentration-dependent manner. (C, E) Neither 8-pMeOPT-2'-O-Me-cAMP nor the two other Epac activators (8-pHPT-2'-O-Me-cAMP and 8-pCPT-2'-O-Me-cAMP) has an effect on InsP_3_-induced Ca^2+ ^release. 8-pCPT-2'-O-Me-cAMP was also ineffective in GTP-containing ICM (lowest two bars). (D, F) All three tested PKA activators (6-Phe-cAMP, 6-BNZ-cAMP, 6-MBC-cAMP) augment InsP_3_-induced Ca^2+ ^release in a concentration-dependent manner. (B, E, F) Means ± S.D., paired t-test, *P < 0.05, **P < 0.01, ***P < 0.001.

The effect of cAMP on InsP_3_-induced Ca^2+ ^release could be mediated by either PKA or Epac. Both target proteins are expressed in blowfly salivary glands [59]. To distinguish between these possibilities, cAMP-analogs that activate either PKA or Epac or both downstream effectors were used instead of cAMP [39]. These cAMP analogs were applied at concentrations of 10 μM and 100 μM. One problem in the quantitative evaluation of these experiments was, that the Mag-fura-2 fluorescence ratio in the β-escin-permeabilized preparations continuously declined as Ca^2+ ^leaked out of the ER (see, for example, Figs. 2A, B; 3A, C, D), and this decline in fluorescence ratio varied between preparations. Therefore, we did not measure and compare the magnitude of Ca^2+ ^release from the ER (as above), but rather its rate as measured by the decline in the Mag-fura-2 fluorescence ratio per minute. The rates were obtained from regression lines fitted to the fluorescence traces over a one minute period before and after application of the cAMP analog (see Fig. 3A, dotted lines). As shown in Figs. 3A and 3B, 8-CPT-cAMP, activating both PKA and Epac, augmented InsP_3_-induced Ca^2+ ^release significantly and in a dose-dependent manner.

Figures 3C–F summarize the effect of three Epac-specific cAMP-analogs and of three PKA-specific analogs on InsP_3_-induced Ca^2+ ^release. At a concentration of 10 μM none of the Epac activators augmented InsP_3_-induced Ca^2+ ^release (Figs. 3C, E). The Epac-activator 8-pHPT-2'-O-Me-cAMP produced a slight but significant increase in the rate of Ca^2+ ^release when applied at a concentration of 100 μM, whereas the other two Epac activators were ineffective at 100 μM. Since Epac links cAMP to the activation of the small G protein Rap1 [9,37] and since our ICM did not contain GTP, we tested whether the above Epac activators were ineffective because of the lack of GTP. However, 8-CPT-O-2'-Me-cAMP had also no significant effect on InsP_3_-induced Ca^2+ ^release when applied in ICM supplemented with 3 mM GTP (Fig. 3E).

In contrast to the Epac activators all tested PKA-specific cAMP analogs augmented InsP_3_-induced Ca^2+^release significantly in a dose-dependent manner (Figs. 3E, F). These findings indicated that the cAMP-dependent augmentation of InsP_3_-induced Ca^2+ ^release was mediated by PKA rather than Epac.

### PKA inhibitors block the augmentation of InsP_3_-induced Ca^2+ ^release by cAMP

**Figure 4:**
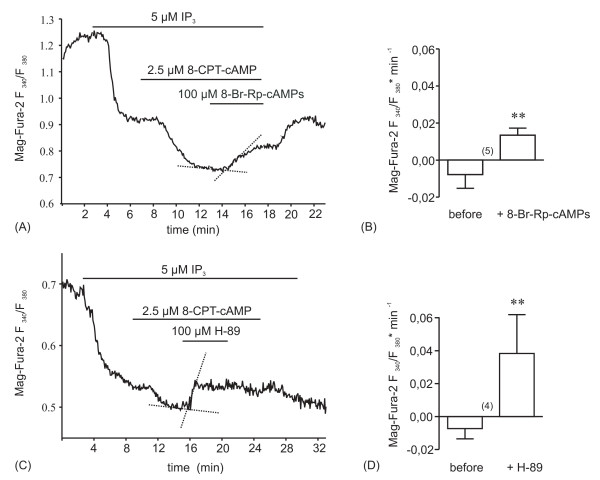
**The competitive antagonist of cAMP-binding to PKA, 8-Br-Rp-cAMPS (A, B), and the PKA inhibitor H-89 (C, D) reverse augmentation of InsP_3_-induced Ca^2+ ^release caused by 8-CPT-cAMP.** Graphs constructed as described for Fig. 3.

To examine by an alternative approach whether the cAMP evoked augmentation of the InsP_3_-induced Ca^2+ ^release was mediated by PKA, we tested the effect of the competitive antagonist of cAMP-binding to PKA, 8-Br-Rp-cAMPS [39,40], and of the PKA inhibitor H-89 [41] on 8-CPT-cAMP-augmented InsP_3_-induced Ca^2+ ^release. Both substances reversed the extra-Ca^2+ ^release produced by 8-CPT-cAMP on a background of 5 μM InsP_3 _(Figs. 4A–D). These results provided further support for our conclusion that the cAMP-evoked augmentation of InsP_3_-induced Ca^2+ ^release was mediated by PKA.

### Does cAMP-mediated augmentation of InsP_3_-induced Ca^2+ ^release affect transepithelial electrolyte transport?

**Figure 5:**
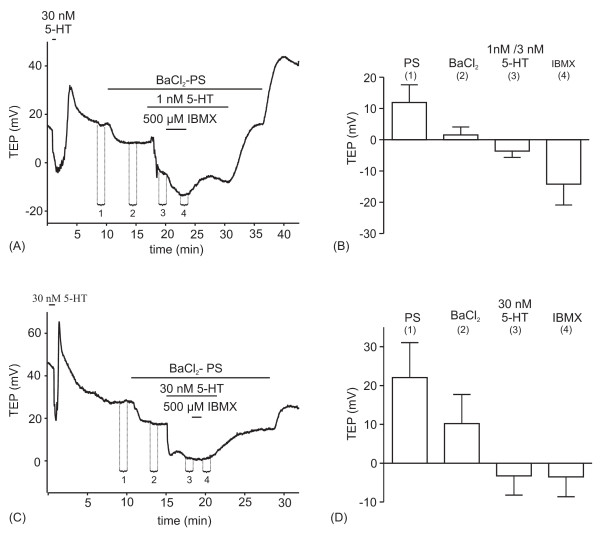
**Effects of IBMX on 5-HT-induced changes in transepithelial potential (TEP) in Ba^2+^-containing PS.** (A, B) Original recordings. The bar graphs (B, D) display and summarize the TEPs recorded at the time points (1–4) as indicated in A and C; means ± S.D. In both groups of experiments (A, C), an initial control stimulation with 30 nM 5-HT produces a biphasic TEP change. The TEP goes negative after superfusion of the preparation with Ba^2+^-containing PS. Addition of 1 nM and 30 nM 5-HT cause the TEP to go further negative. The TEP recorded in the presence of 1 nM 5-HT (A, B) but not 30 nM 5-HT (C, D) goes further negative by application of 500 μM IBMX in the presence of 5-HT.

The transepithelial potential (TEP) is a sensitive indicator of the transepithelial K^+ ^and Cl^- ^transport that results from 5-HT-induced activation of the InsP_3_/Ca^2+ ^and cAMP signaling pathways, because K^+ ^transport is activated by cAMP and Cl^- ^transport is activated by Ca^2+ ^[34,38]. We used TEP measurements in order to examine whether cAMP was able to amplify transepithelial Cl^- ^transport induced (1) by 5-HT concentrations that were just sufficient to stimulate fluid secretion and (2) by saturating 5-HT concentrations. Because cAMP also stimulates transepithelial K^+ ^transport by activating an apical vacuolar-type H^+^-ATPase that energizes K^+ ^transport [33,42,43], we had to minimize the contribution of transepithelial K^+ ^transport to 5-HT-induced TEP changes. This was accomplished by using a K^+^-free PS containing 7.5 mM of the K^+ ^channel blocker Ba^2+ ^to block basolateral K^+ ^entry [44], as illustrated in Fig. 5A. A brief control stimulation with 30 nM 5-HT produced a biphasic change of the TEP. The negative-going phase of the TEP change was attributable to transepithelial Cl^- ^transport, and the positive-going phase was caused by the somewhat delayed transepithelial K^+ ^transport [34]. Superfusion of the preparation with BaCl_2_-containig PS caused the TEP to become negative by about 10 mV, because the resting TEP was slightly positive attributable to some transepithelial K^+ ^transport in the unstimulated gland. Upon application of 1 nM 5-HT to the BaCl_2_-containing PS, the TEP became more negative (Fig. 5A), as a result of 5-HT-induced Ca^2+ ^release [26] and a Ca^2+^-induced activation of transepithelial Cl^- ^transport. Most significantly, 500 μM IBMX caused the TEP to become even more negative in the presence of 1 nM 5-HT. The effects of IBMX, 5-HT, and Ba^2+ ^were reversible. Fig. 5B summarizes the results of several experiments of this kind and displays the TEP recorded at four selected time points indicated in Fig. 5A. The experiment illustrated in Fig. 5C is identical, except that the preparation was stimulated with 30 nM 5-HT, a concentration that saturates the rate of fluid transport. At this high 5-HT concentration, IBMX caused no further change of the TEP (Fig. 5D).

The results of these TEP measurements indicate that an increase in intracellular cAMP concentration (by application of the phosphodiesterase inhibitor IBMX) augments the effect of a threshold concentration of 5-HT on transepithelial Cl^- ^transport. This result is in agreement with above finding that cAMP sensitizes the InsP_3_R Ca^2+ ^channel for InsP_3_. The physiological consequence of InsP_3_R sensitization is measurable only when the glands are stimulated by low 5-HT concentrations.

## Discussion

The results of this study provide physiological evidence that cAMP augments InsP_3_-induced Ca^2+ ^release from the ER in the salivary glands of *Calliphora vicina*, a dipteran fly closely related to *Drosophila melanogaster*. Our intraluminal Ca^2+ ^measurements in the ER of permeabilized cells in isolated glands show, in addition, that cAMP increases the affinity of the InsP_3_R for InsP_3 _by about a factor of 10. Using cAMP analogs that activate either PKA or Epac and PKA inhibitors we show further that this cAMP effect is mediated by PKA rather than Epac. Finally, intracellular Ca^2+ ^measurements and electrophysiological recordings indicate that the cAMP-induced and PKA-mediated sensitization of the InsP_3_R for InsP_3 _affects Ca^2+ ^signaling and transepithelial electrolyte transport.

### cAMP-induced and PKA-mediated augmentation of InsP_3_-induced Ca^2+ ^release

All three mammalian InsP_3_R subtypes have the potential to undergo phosphorylation by PKA and by some other kinases including PKG, PKC and CaM-kinase [22,45]. The resulting phosphoregulation of Ca^2+ ^release is thought to have profound effects on the spatio-temporal characteristics of Ca^2+ ^signals and to provide a potential mechanism of crosstalk between different signaling pathways. Nevertheless, data on the effects of InsP_3_R phosphorylation on InsP_3_-induced Ca^2+ ^release are contradictory (reviewed in [1,46]). Most reports suggest that InsP_3_R phosphorylation augments InsP_3_-induced Ca^2+ ^release (e.g. [12,15,17,47-49]], whereas others indicate that Ca^2+ ^release is attenuated [e.g. [14,50]].

Here, we show that cAMP augments InsP_3_-induced Ca^2+ ^release in permeabilized salivary glands of *Calliphora*, and that the effect of cAMP is mediated by PKA. The cAMP-dependent leftward shift in the dose-response relationship for InsP_3 _suggests that the augmentation of Ca^2+ ^release is attributable to an increase of about 10-fold in the affinity of the InsP_3_R Ca^2+ ^channel for InsP_3_. We can exclude the possibility that the cAMP-induced augmentation of Ca^2+ ^release results from a stimulation of Ca^2+ ^loading of the ER via SERCA, because the intraluminal Ca^2+ ^concentration is not affected by cAMP-containing ICM in the permeabilized glands.

The involvement of PKA suggests that the cAMP effect is mediated by phosphorylation of InsP_3_R. However, although six potential PKA phosphorylation sites have been detected in the sequence of *Caenorhabditis elegans *InsP_3_R, no such sites have been identified in *Drosophila melanogaster *InsP_3_R (DmInsP_3_R) [19,21,22]. It must be noted, however, that only a single algorithm had been used to search for putative sites for PKA-mediated phosphorylation in the *Drosophila *InsP_3 _receptor. We experienced that, at least for other proteins, results for putative phosphorylation sites vary by using different bioinformatic algorithms [Voss et al., 2007]. Sequence information for *Calliphora *InsP_3_R is still lacking but the dipteran fly *Calliphora *is closely related to *Drosophila*. Thus, whether fly InsP_3 _receptor Ca^2+ ^channels can be phosphorylated, or whether the InsP_3_R in *Calliphora *differs from that in *Drosophila *with respect to consensus sites for PKA-mediated phosphorylation remains unknown. Therefore, we cannot yet explain the molecular basis of the cAMP-induced and PKA-mediated sensitization of Ca^2+ ^release in this species. DmInsP_3_R seems to have consensus sequences for phosphorylation by PKC and CaM-kinase II [21]. The activity of these two kinases can be affected by PKA [17,51-53]. Thus, cAMP might affect DmInsP_3_R via other kinases or unknown accessory proteins that are phosphorylated by PKA.

### Physiological consequences of cAMP-mediated sensitization of the InsP3R for InsP_3_R for InsP_3_

The cAMP-mediated sensitization of the InsP_3_R for InsP_3 _has measurable effects on Ca^2+ ^signaling in *Calliphora *salivary glands. We have shown that increasing the intracellular cAMP concentration converts baseline Ca^2+ ^spiking induced by threshold concentrations of 5-HT [26] into a sustained Ca^2+ ^elevation. This effect of cAMP on Ca^2+ ^spiking is remarkably similar to that reported for the parotid acinar cell. Here, forskolin potentiates carbachol-induced [Ca^2+^]_i _changes, and this potentiation also results from enhanced Ca^2+ ^release attributable to cAMP-dependent and PKA-mediated potentiation of InsP_3_-induced Ca^2+ ^release from the ER [17]. The enhanced Ca^2+ ^release is probably not the result of a cAMP-dependent stimulation of InsP_3 _production [17], although cAMP has been shown to potentiate InsP_3 _production in hepatocytes and parotid acinar cells [54,55]. This possibility can be excluded in *Calliphora *salivary glands, as IBMX, although it potentiates 5-HT-induced fluid secretion (see below), has no effect on 5-HT-induced [^3^H]inositol release from isolated glands [56]. Thus, in *Calliphora *salivary glands, in parotid salivary glands, and in a number of other secretory cell types (such as pancreatic β cells), the InsP_3_R Ca^2+ ^channel obviously functions as a coincidence detector [18] that monitors a simultaneous increase of InsP_3_, cAMP, and Ca^2+ ^concentrations, the last-mentioned because InsP_3_R is also regulated by Ca^2+ ^[reviewed in [22]].

Recordings of the transepithelial potential (TEP) in *Calliphora *salivary glands indicate that cAMP also augments the Ca^2+^-dependent transepithelial Cl^- ^transport induced by low 5-HT concentrations, an observation suggesting that the cAMP-dependent enhanced Ca^2+ ^release additionally affects fluid secretion. This notion is supported by experiments dating back more than 30 years. In the early 1970s, Berridge [57,58] found that the phosphodiesterase inhibitor theophylline sensitized 5-HT-induced fluid secretion from *Calliphora *salivary glands by a factor of about 10.

## Conclusion

Taking all these data together, we can now ascribe two physiological effects to cAMP in *Calliphora *salivary glands: (1) the activation of an apical vacuolar-type H^+^-ATPase [33,59] that energizes the apical membrane for *n*H^+^/K^+^-antiporter-mediated K^+ ^transport, and (2) the augmentation of InsP_3_-induced Ca^2+ ^release from the ER resulting in enhanced Ca^2+ ^signaling and enhanced transepithelial Cl^- ^transport and fluid secretion. Both actions of cAMP are mediated by PKA, which is present at the sites of these effector proteins, the ER, and the apical membrane [59].

## Methods

### Animals, preparation and solutions

The blowfly, *Calliphora vicina*, was reared at our Institute. Flies were kept at 24–26°C under a 12 h light: 12 h dark cycle. The abdominal region of the tubular salivary glands of adult flies was dissected under physiological solution (PS).

Normal PS contained (mM): 128 NaCl, 10 KCl, 2 CaCl_2_, 2 MgCl_2_, 2.8 maleic acid, 3 sodium glutamate, 10 TRIS-HCl, 10 D-Glucose, pH 7.2. Ca^2+^-free PS was prepared by omitting CaCl_2 _and adding 2 mM EGTA. "Intracellular-like" medium (ICM) was used for experiments with β-escin-permeabilized preparations and contained (mM): 125 KCl, 20 NaCl, 2 MgCl_2_, 3 Na_2_ATP, 0.1 EGTA, 0.06 CaCl_2_, 10 HEPES at pH 7.3. The free Ca^2+ ^concentration in this medium was determined to be ~250 nM, as noted previously [32]. GTP-ICM contained (mM): 125 KCl, 20 NaCl, 2 MgCl_2_, 3 Na_2_ATP, 3 GTP, 0.1 EGTA, 0.06 CaCl_2_, 10 HEPES at pH 7.3.

### Transepithelial potential recordings

Because the transepithelial potential (TEP) is a sensitive indicator of the transepithelial K^+ ^and Cl^- ^transport [28,34,38], we used TEP recordings to obtain information about the effects of cAMP on transepithelial Cl^- ^transport that is activated by an increase in intracellular Ca^2+ ^concentration. Isolated salivary gland tubules (ca. 10 mm long) were placed across a narrow paraffin oil gap into a two-well perfusion chamber that was modified according to [28]. One well contained the closed end of the gland tubule and was continuously perfused with PS. The cut end of the salivary gland opened into the other well. Both wells were connected via 3 M KCl agar-bridges and AgAgCl-pellets in microelectrode holders (WPI Int., Berlin, Germany) to a differential amplifier (npi-electronics, Tamm, Germany). Data were sampled and digitized at 2 Hz (A/D-board: DAS-1600; Keithley, Germering, Germany). The software EASYEST (Asyst Software Technologies Inc., Rochester, NY) was used for data acquisition and storage, and SigmaPlot 8.0 software for offline data analysis.

### Dye loading and cell permeabilization

For intracellular Ca^2+ ^measurements the dissected glands were loaded with fura-2 by incubation with 5 μM fura-2 acetoxymethylester in PS for 40–60 min at room temperature. After dye loading, the gland tubules were mounted on cover slips coated with VectaBond™ (Axxora, Grünberg, Germany) and placed in a superfusion chamber on the stage of a Zeiss Axiovert 135TV epifluorescence microscope. In all experiments, the preparations were continuously superfused with PS (or with Ca^2+^-free PS) at a rate of ~1 ml/min.

For intraluminal Ca^2+ ^measurements in the ER the glands were loaded with mag-fura-2 by a 20 min incubation with 1 μM mag-fura-2 AM in PS and subsequently mounted in glass-bottomed perfusion chambers as described above. The glands were then permeabilized for 4–8 min in ICM containing 200 μg ml^-1 ^(w/v) β-escin. After permeabilization, excessive β-escin was washed out with ICM. The progress of permeabilization was monitored by following the decrease in mag-fura-2 fluorescence until the signal had reached a stable level attributable to the loss of cytosolic dye.

### Measurements of [Ca^2+^]_i_

[Ca^2+^]_i _was measured as described previously [26]. In brief, pairs of fluorescence images, excited at wavelengths of 340 nm and 380 nm (VisiChrome High Speed Polychromator System; Visitron Systems, Puchheim, Germany) via a 450 nm dichroic mirror and a Zeiss Fluar 20/0.75 objective, were captured at a rate of 1 Hz with a cooled frame transfer CCD camera (TE/CCD-512EFT; Princeton Instruments Corp., Trenton, NJ) via a 515–565 nm bandpass filter. Raw images were processed on a PC by using the software MetaFluor (Universal Imaging Corp., West Chester, PA). Fluorescence ratios (340 nm/380 nm) were calculated after subtraction of background fluorescence and cell autofluorescence both of which were determined at the end of every experiment by quenching fura-2 fluorescence by application of 20 mM MnCl_2_.

### Statistical analysis

Signal processing and curve fitting were performed by using GraphPad Prism 4 (Version 4.01, GraphPad Software Inc.). Data are expressed as means ± S.D. Statistical comparisons were made by a Student's paired *t*-test, and *P *values < 0.05 were considered significant.

## Authors' contributions

RS carried out all experiments and drafted the manuscript. BW and OB participated in the conception of the project and the design of the experiments, and they helped to write the manuscript. All authors approved the final manuscript.

## References

[B1] Bruce JIE, Straub SV, Yule DI (2003). Crosstalk between cAMP and Ca^2+ ^signaling in non-excitable cells. Cell Calcium.

[B2] Zaccolo M, Pozzan T (2003). cAMP and Ca^2+ ^interplay: a matter of oscillation. TINS.

[B3] Berridge MJ, Lipp P, Bootman MD (2000). The versatility and universality of calcium signalling. Nature Rev Molec Cell Biol.

[B4] Fewtrell C (1993). Ca^2+ ^oscillations in non-excitable cells. Annu Rev Physiol.

[B5] Petersen OH, Michalak M, Verkhratsky A (2005). Calcium signalling: past, present and future. Cell Calcium.

[B6] Misaki N, Imaizumi TY, Watanabe Y (1989). Cyclic AMP-dependent protein kinase interferes with GTP gamma S stimulated IP_3 _formation in differentiated HL-60 cell membranes. Life Sci.

[B7] Wu D, Katz A, Simon MI (2001). Activation of phospholipase C β_2 _by the α and βγ subunits of trimeric GTP-binding protein. Proc Natl Acad Sci USA.

[B8] Kennedy CR, Proulx PR, Hebert RL (1995). Regulation of bradykinin-stimulated phospholipase C and arachidonic acid release by protein kinase A in MDCK-D1 cells. Biochim Biophys Acta.

[B9] de Rooij J, Zwartkruis FJ, Verheijen MH, Cool RH, Nijman SM, Wittinhofer A, Bos JL (1998). Epac is a Rap1 guanone-mucleotide-exchange factor directly activated by cyclic AMP. Nature.

[B10] Bos JL (2003). Epac: a new cAMP target and new avenues in cAMP research. Nat Rev Mol Cell Biol.

[B11] Ferris CD, Cameron AM, Bredt DS, Huganit RL, Snyder SH (1991). Inositol 1,4,5-trisphosphate receptor is phosphorylated by cyclic AMP-dependent protein kinase at serins 1755 and 1589. Biochem Biophys Res Commun.

[B12] Hajnoczky G, Gao E, Nomura T, Hoek JB, Thomas AP (1993). Multiple mechanisms by which protein kinase A potentiates inositol 1,4,5-trisphosphate-induced Ca^2+ ^mobilization in permeabilized hepatocytes. Biochem J.

[B13] Nakade S, Rhee SK, Hamanaka H, Mikoshiba K (1994). Cyclic AMP-dependent phosphorylation of an immunoaffinity-purified homotetrameric inositol 1,4,5-trisphosphate receptor (type I) increases Ca^2+ ^flux in reconstituted lipid vesicles. J Biol Chem.

[B14] Tertyshnikova S, Fein A (1998). Inhibition of inositol 1,4,5-trisphosphate-induced Ca^2+ ^release by cAMP-dependent protein kinase in a living cell. Proc Natl Acad Sci USA.

[B15] Wojcikiewicz RJ, Luo SG (1998). Phosphorylation of inositol 1,4,5-trisphosphate receptors by cAMP-dependent protein kinase. Type I, II, and III receptors are differentially susceptible to phosphorylation and are phosphorylated in intact cells. J Biol Chem.

[B16] Giovannucci DR, Groblewski GE, Sneyd J, Yule DI (2000). Target phosphorylation of inositol 1,4,5-trisphosphate receptors selectively inhibit localized Ca^2+ ^release and shapes oscillatory Ca^2+ ^signals. J Biol Chem.

[B17] Bruce JIE, Shuttleworth TJ, Giovannucci DR, Yule DI (2002). Phosphorylation of inositol 1,4,5-trisphosphate receptors in parotid acinar cells. J Biol Chem.

[B18] Kang G, Chepurny OG, Rindler MJ, Collis L, Chepurny Z, Li WH, Harbeck M, Roe MW, Holz GG (2005). A cAMP and Ca^2+ ^coincidence detector in support of Ca^2+^-induced Ca^2+ ^release in mouse pancreatic β cells. J Physiol (Lond).

[B19] Venkatesh K, Siddharta G, Joshi R, Pate SL, Hasan G (2001). Interactions between inositol 1,4,5-trisphosphate and cAMP signaling pathways regulate larval molting in *Drosophila*. Genetics.

[B20] Hasan G, Rosbash M (1992). *Drosophila *homologs of two mammalian intracellular Ca^2+^-release channels: identification and expression patterns of inositol 1,4,5-trisphosphate and the ryanodine receptor genes. Development.

[B21] Yoshikawa S, Tanimura T, Miyawaki A, Nakamura M, Yuzaki M, Furuichi T, Mikoshiba K (1992). Molecular cloning and characterization of the inositol 1,4,5-trisphosphate receptor in *Drosophila melanogaster*. J Biol Chem.

[B22] Bezprozvanny I (2005). The inositol 1,4,5-trisphosphate receptors. Cell Calcium.

[B23] Srikanth S, Wang Z, Tu H, Nair S, Mathew MK, Hasan G, Bezprozvanny I (2004). Functional properties of the *Drosophila melanogaster *inositol 1,4,5-trisphosphate receptor mutants. Biophys J.

[B24] Swatton JE, Morris SA, Wissing F, Taylor CW (2001). Functional properties of *Drosophila *inositol trisphosphate receptors. Biochem J.

[B25] Berridge MJ (2005). the secrets of cell signaling. Annu Rev Physiol.

[B26] Zimmermann B, Walz B (1997). Serotonin-induced intercellular calcium waves in salivary glands of the blowfly *Calliphora erythrocephala*. J Physiol (Lond).

[B27] Zimmermann B, Walz B (1999). The mechanism mediating regenerative intercellular Ca^2+ ^waves in the blowfly salivary gland. EMBO J.

[B28] Berridge MJ, Prince WT (1972). Transepithelial potential changes during stimulation of isolated salivary glands with 5-hydroxytryptamine and cyclic AMP. J Exp Biol.

[B29] Prince WT, Berridge MJ (1972). The effects of 5-hydroxytryptamine and cyclic AMP on the potential profile across isolated salivary glands. J Exp Biol.

[B30] Berridge MJ, Lindley BD, Prince WT (1975). Membrane permeability changes during stimulation of isolated salivary glands of *Calliphora *by 5-hydroxytryptamine. J Physiol (Lond).

[B31] Berridge MJ, Lindley BD, Prince WT (1976). Studies on the mechanisms of fluid secretion by isolated salivary glands of *Calliphora*. J Exp Biol.

[B32] Zimmermann B (2000). Control of InsP_3_-induced Ca^2+ ^oscillations in permeabilized blowfly salivary gland cells: contribution of mitochondria. J Physiol (Lond).

[B33] Dames P, Zimmermann B, Schmidt R, Rein J, Voss M, Schewe B, Walz B, Baumann O (2006). cAMP regulates plasma membrane vacuolar-type H^+^-ATPase assembly and activity in blowfly salivary glands. Proc Natl Acad Sci USA.

[B34] Berridge MJ (1970). The role of 5-hydroxytryptamine and cyclic AMP in the control of fluid secretion by isolated salivary glands. J Exp Biol.

[B35] Hofer AM, Machen TE (1994). Direct measurements of free Ca^2+ ^in organelles of gastric epithelial cells. Am J Physiol.

[B36] Chatton JY, Liu H, Stucki J (1995). Simultaneous measurements of Ca^2+ ^in the intracellular stores and the cytosol of hepatocytes during hormone-induced Ca^2+ ^oscillations. FEBS Lett.

[B37] Kawasaki H, Springett GMN, Mochizuki N, Toki S, Nakaya M, Matsuda M, Housman DE, Graybiel AM (1998). A family of cAMP-binding proteins that directly activate Rap1. Science.

[B38] Berridge MJ, Patel NG (1968). Insect salivary glands: stimulation of fluid secretion by 5-hydroxytryptamine and adenosine-3',5'-monophosphate. Science.

[B39] Christensen AE, Selheim F, de Rooij J, Dremier S, Schwede F, Dao KK, Martinez A, Maenhaut C, Bos JL, Genieser HG, Doskeland SO (2003). cAMP analog mapping of Epac1 and cAMP kinase. Discriminating analogs demonstrate that Epac and cAMP kinase act synergistically to promote PC-12 cell neurite extension. J Biol Chem.

[B40] Gjertsen BT, Mellgren G, Otten A, Maronde E, Genieser HG, Jastorff B, Vintermyr OK, McKnight GS, Doskeland SO (1995). Novel (Rp)-cAMPS analogs as tools for inhibition of cAMP-kinase in cell culture. Basal cAMP-kinase activity modulates interleukin-1 beta action. J Biol Chem.

[B41] Chijiwa T, Mishima A, Hagiwara M, Sano M, Hayashi K, Inoue T, Nairo K, Toshioka T, Hidaka H (1990). Inhibition of forskolin-induced neurite outgrowth and protein phosphorylation by a newly synthesized selective inhibitor of cyclic AMP-dependent protein kinase, N-[2-(p-bromocinnamylamino)ethyl]-5-isoquinolinesulfonamide (H-89), of PC12D pheochromocytoma cells. J Biol Chem.

[B42] Zimmermann BP, Dames PB, Walz BO, Baumann O (2003). Distribution and serotonin-induced activation of vacuolar-type H^+^-ATPase in the salivary glands of the blowfly *Calliphora vicina*. J Exp Biol.

[B43] Rein J, Zimmermann B, Hille C, Lang I, Walz B, Baumann O (2006). Fluorescence measurements of serotonin-induced V-ATPase-dependent pH changes at the luminal surface in salivary glands of the blowfly *Calliphora vicina*. J Exp Biol.

[B44] Ianowski JP, O'Donnell MJ (2004). Basolateral ion transport mechanisms during fluid secretion by *Drosophila *Malpighian tubules: Na^+^:K^+^:2Cl^- ^cotransport and Cl^- ^conductance. J Exp Biol.

[B45] Yule DI, Straub SV, Bruce JIE (2003). Modulation of Ca^2+ ^oscillations by phosphorylation of Ins(1,4,5)P_3 _receptors. Biochem Soc Trans.

[B46] Straub SV, Wagner LE, Bruce JIE, Yule DI (2004). Modulation of cytosolic calcium signaling by protein kinase A-mediated phosphorylation of inositol 1,4,5-trisphosphate receptors. Biol Res.

[B47] Brown DA, Bruce JIE, Straub SV, Yule DI (2004). cAMP potentiates ATP-evoked calcium signaling in human parotid acinar cells. J Biol Chem.

[B48] Dyachok O, Gylfe E (2004). Ca^2+^-induced Ca^2+ ^release via inositol 1,4,5-trisphosphate receptors is amplified by protein kinase A and triggers exocytosis in pancreatic beta-cells. J Biol Chem.

[B49] Joseph SK, Ryan SV (1993). Phosphorylation of the inositol trisphosphate receptor in isolated rat hepatocytes. J Biol Chem.

[B50] Volpe P, Alderson-Lang BH (1990). Regulation of inositol 1,4,5-trisphosphate-induced Ca^2+ ^release. II. Effect of cAMP-dependent protein kinase. Am J Physiol.

[B51] Daaka Y, Luttrell LM, Lefkowitz RJ (1997). Switching of the coupling of the beta2-adrenergic receptor to different G-proteins by protein kinase A. Nature.

[B52] Wayman GA, Tokumitsu H, Soderling TR (1997). Inhibitory crosstalk by cAMP kinase on the calmodulin-dependent protein kinase cascade. J Biol Chem.

[B53] Valverde RH, Tortelote GG, Lemos T, Mintz E, Vieyra A (2005). Ca^2+^/calmodulin-dependent protein kinase II is an essential mediator in the coordinated regulation of electrocyte Ca^2+^-ATPase by calmodulin and protein kinase A. J Biol Chem.

[B54] Pittner RA, Fain JN (1989). Exposure of cultured hepatocytes to cyclic AMP enhances the vasopressin-mediated stimulation of inositol phosphate production. Biochem J.

[B55] Horn VJ, Baum BJ, Ambudkar IS (1988). Beta-adrenergic receptor stimulation induces inositol trisphosphate production and Ca^2+ ^mobilization in rat parotid acinar cells. J Biol Chem.

[B56] Fain JN, Berridge MJ (1979). Relationship between hormonal activation of phosphatidyl hydrolysis, fluid secretion and calcium flux in the blowfly salivary gland. Biochem J.

[B57] Berridge MJ (1970). The role of 5-hydroxytryptamine and cyclic AMP in the control of fluid secretion by isolated salivary glands. J Exp Biol.

[B58] Berridge MJ (1972). Transepithelial potential changes during stimulation of isolated salivary glands with 5-hydroxytryptamine and cyclic AMP. J Exp Biol.

[B59] Rein J, Voss M, Blenau W, Walz B, Baumann O (2008). Hormone-induced assembly and activation of V-ATPase in blowfly salivary glands is mediated by protein kinase A. Am J Physiol Cell Physiol.

